# Vascular response patterns to targeted therapies in murine breast cancer models with divergent degrees of malignancy

**DOI:** 10.1186/s13058-023-01658-9

**Published:** 2023-05-23

**Authors:** Emily Hoffmann, Mirjam Gerwing, Tobias Krähling, Uwe Hansen, Katharina Kronenberg, Max Masthoff, Christiane Geyer, Carsten Höltke, Lydia Wachsmuth, Regina Schinner, Verena Hoerr, Walter Heindel, Uwe Karst, Michel Eisenblätter, Bastian Maus, Anne Helfen, Cornelius Faber, Moritz Wildgruber

**Affiliations:** 1 Clinic of Radiology, University of Münster, Münster, Germany; 2 Institute for Musculoskeletal Medicine, University of Münster, Münster, Germany; 3 Institute of Inorganic and Analytical Chemistry, University of Münster, Münster, Germany; 4 Department of Radiology, University Hospital, LMU Munich, Munich, Germany; 5 Heart Center Bonn, Department of Internal Medicine II, University Hospital Bonn, Bonn, Germany; 6 Department of Diagnostic and Interventional Radiology, Medical Faculty OWL, University of Bielefeld, Bielefeld, Germany

**Keywords:** Tumor vasculature, Magnetic resonance imaging, Targeted therapy, Tumor microenvironment, Immune checkpoint inhibition, Sorafenib

## Abstract

**Background:**

Response assessment of targeted cancer therapies is becoming increasingly challenging, as it is not adequately assessable with conventional morphological and volumetric analyses of tumor lesions. The tumor microenvironment is particularly constituted by tumor vasculature which is altered by various targeted therapies. The aim of this study was to noninvasively assess changes in tumor perfusion and vessel permeability after targeted therapy in murine models of breast cancer with divergent degrees of malignancy.

**Methods:**

Low malignant 67NR or highly malignant 4T1 tumor-bearing mice were treated with either the multi-kinase inhibitor sorafenib or immune checkpoint inhibitors (ICI, combination of anti-PD1 and anti-CTLA4). Dynamic contrast-enhanced magnetic resonance imaging (DCE-MRI) with i.v. injection of albumin-binding gadofosveset was conducted on a 9.4 T small animal MRI. Ex vivo validation of MRI results was achieved by transmission electron microscopy, immunohistochemistry and laser ablation-inductively coupled plasma-mass spectrometry.

**Results:**

Therapy-induced changes in tumor vasculature differed between low and highly malignant tumors. Sorafenib treatment led to decreased tumor perfusion and endothelial permeability in low malignant 67NR tumors. In contrast, highly malignant 4T1 tumors demonstrated characteristics of a transient window of vascular normalization with an increase in tumor perfusion and permeability early after therapy initiation, followed by decreased perfusion and permeability parameters. In the low malignant 67NR model, ICI treatment also mediated vessel-stabilizing effects with decreased tumor perfusion and permeability, while ICI-treated 4T1 tumors exhibited increasing tumor perfusion with excessive vascular leakage.

**Conclusion:**

DCE-MRI enables noninvasive assessment of early changes in tumor vasculature after targeted therapies, revealing different response patterns between tumors with divergent degrees of malignancy. DCE-derived tumor perfusion and permeability parameters may serve as vascular biomarkers that allow for repetitive examination of response to antiangiogenic treatment or immunotherapy.

**Supplementary Information:**

The online version contains supplementary material available at 10.1186/s13058-023-01658-9.

## Background

Since angiogenesis has been identified as one hallmark of cancer [1], the role of tumor vasculature in tumorigenesis is of prime interest. Angiogenesis is defined as the formation of new blood vessels from a pre-existing vascular bed, driven by predominance of proangiogenic factors and corresponding signaling pathways [2, 3]. In contrast to physiological blood vessel formation, which is an organized and controlled process, rapid tumor growth causes angiogenesis in tumor tissue to be dysregulated, leading to immature tumor vasculature [2] with several structural and functional abnormalities. Tumor-associated blood vessels are characterized by variable diameters and distorted shapes [3–5]. The endothelial layer is often tortuous and disrupted, leading to enhanced permeability with increased interstitial pressure [2, 5]. While these characteristics promote tumor dissemination to distant organs, they also lead to treatment limitations by restricted drug delivery into the tumor lesion and consequently reduced therapeutic effectiveness [5–7].

Many targeted cancer therapies closely interact with the tumor vasculature. To specifically target the aberrant properties of tumor vasculature itself, a variety of antiangiogenic therapies have been developed, mainly addressing the vascular endothelial growth factor pathway as one of the most significant proangiogenic factors [8]. By restoring the balance between pro- and antiangiogenic pathways, antiangiogenic therapies aim for structural stabilization of the blood vessel wall, mediated by establishment of inter-endothelial contacts, re-development of regularly structured basement membranes and association and integration of vascular mural cells [9, 10]. This structural stabilization with subsequently reduced vascular permeability contributes to vascular normalization, the overall process of remodeling tumor blood vessels to restore their structure and function, and thereby leading to improved blood supply and reduction in intratumoral hypoxia [11].

In addition to antiangiogenic therapies, other classes of targeted therapies, especially immunotherapies, are also closely coupled to the vascular system [12]. Since tumor blood vessels constitute a component of the tumor microenvironment (TME), there is a complex bidirectional interplay between tumor vasculature and immune cells. On the one hand, characteristics of tumor vasculature, e.g., endothelial permeability and expression of adhesion molecules, influence trafficking and migration of immune cells into the tumor lesion [13, 14]. On the other hand, activated immune cells within the TME, especially tumor-associated macrophages, neutrophils and T-cells, can produce additional angiogenic factors with subsequent effects on tumor perfusion and permeability [15].

Even though these targeted therapies are approved for a wide range of solid tumors [5, 16], including breast cancer [17], their effectiveness is limited to only a subgroup of patients while others show innate or acquired resistances [18, 19]. This variable response is presumably based on pronounced intra- and intertumoral heterogeneity of the TME. In this context, varying distribution in tumor vasculature within a single tumor lesion itself or between different cancer patients might lead to variable therapeutic drug delivery [20, 21]. Thus, as there is a significant number of patients who do not benefit from targeted therapies, early and repeated examination of therapy response is needed.

A noninvasive imaging approach to investigate the characteristics of tumor vasculature, a main component of the TME that is altered by targeted therapies, is constituted by dynamic contrast-enhanced magnetic resonance imaging (DCE-MRI). By obtaining fast MRI sequences before, during and after rapid intravenous administration of gadolinium-based contrast agents and subsequent pharmacokinetic modeling, DCE-MRI allows for quantification of tumor perfusion and permeability [22]. This MRI technique has already been applied to assess response to antiangiogenic treatment both in preclinical [23, 24] and clinical settings [25].

Here, DCE-MRI was performed to compare the vascular response patterns between low malignant 67NR and highly malignant 4T1 murine breast cancer models to different classes of targeted therapies. First, the sensitivity of the applied MR imaging approach to dynamically capture specific modulation of tumor vasculature characteristics was tested. To this end, dedicated manipulation of vasculature features was conducted by treating tumors with angiopoietin-1, which induces stabilization of blood vessel walls [26]. Then, treatment with targeted therapies, including either a tyrosine kinase inhibitor or immune checkpoint inhibitors (ICI), was performed. While sorafenib is a multi-kinase inhibitor directly influencing tumor vasculature by inhibiting angiogenesis [27], immune checkpoint inhibitors primarily address immune cells and tumor-promoting inflammation with secondary effects on tumor vasculature [28]. MRI results were confirmed by ex vivo analysis of tumor vasculature, including histology, immunohistochemistry and transmission electron microscopy.

## Materials and methods

### Mouse models

All animal experiments were approved by the responsible authorities (Landesamt für Natur, Umwelt und Verbraucherschutz NRW, Protocol No. 81-02.04.2018.A010). A total of 111 female BALB/c mice (Charles River Laboratories, Sulzberg, Germany) were used at the age of eight to twelve weeks and were kept under a 12 h light–dark-cycle, provided with food and water ad libitum. Two syngeneic murine breast cancer models have been used: highly malignant 4T1 tumors, metastasizing in regional lymph nodes and distant organs, primarily the lung, liver and bones, and 67NR tumors that grow noninvasively and do not develop metastases [29, 30]. Primarily based on their different growth kinetics, both tumor models contain significant differences in tumor vasculature. Due to rapid tumor growth, highly malignant 4T1 tumors show distorted blood vessels with a thin and disrupted endothelial layer. In contrast, slower growth of low malignant 67NR tumors enables intratumoral neoangiogenesis to proceed in a more controlled manner, resulting in increased maturity and integrity of intratumoral blood vessels [31].

### Cell culture

4T1 and 67NR cells were cultured in Dulbecco’s modified Eagle’s medium (DMEM, Thermo Fisher Scientific, Waltham, Massachusetts, USA), supplemented with 10% fetal bovine serum in standard conditions (37 °C, 5% CO_2_).

### Tumor implantation

For tumor implantation, 10^6^ 4T1 or 67NR cells, resuspended in 25 µL cell culture medium, were implanted orthotopically into the lower left mammary fat pad. For daily scoring of the mice, tumor sizes were measured daily using a digital caliper.

### Angiopoietin-1-induced vessel stabilization

To test whether the MR imaging approach is sensitive enough to detect targeted manipulation of tumor vasculature, 4T1 tumor-bearing mice were treated with angiopoieitin-1 that is known to promote vessel stabilization [26]. Starting the day after tumor implantation, angiopoietin-1 treatment was administered by tail vein injection (5 µg/kg) every day [32]. MR imaging was conducted on day six after tumor implantation. Afterward, mice were killed and tumors were prepared for ex vivo analysis.

### Targeted therapies

Treatment initiation of both targeted therapies (sorafenib or immune checkpoint inhibitors) was started on day three after tumor implantation. Sorafenib treatment (Selleck Chemicals, Houston, Texas, USA) was given as a daily oral gavage (30 mg/kg) [33]. Besides a weight loss of up to 5% during therapy, mice tolerated treatment well. As combination treatment enhances efficient anticancer activity, especially in 4T1 tumors [34], immune checkpoint blockade was induced by a combination of anti-CTLA4 and anti-PD1 inhibition (BioXCell, Lebanon, USA) via i.p. injection (10 mg/kg of each drug) every second day [35]. Mice tolerated treatment well without any observable side effects. Data were compared to untreated control groups that received no therapy after tumor implantation. MR imaging was conducted on two sets of animals on either day six or day nine, and after MRI scans, mice were killed and tumors were prepared for ex vivo analysis.

### MR imaging and analysis

MR imaging was performed on a 9.4 T small animal BioSpec system (Bruker BioSpin GmbH, Ettlingen, Germany), equipped with a ^1^H quadrature volume resonator for signal excitation and a 10 mm surface coil for signal reception (Bruker), using ParaVision 6.0.1. Mice were anesthetized with 1.5% isoflurane in 1.5 L/min of oxygen and compressed air (1:4) under continuous respiratory and temperature monitoring. Mice were placed in supine position and for reduction in susceptibility artifacts, the area between tumor and surface coil was covered in alginate (Johannes Weithas, Lütjenburg, Germany). For anatomical information, a T2-weighted multi-slice RARE (Rapid Acquisition with Relaxation Enhancement) sequence (TR = 2500 ms, TE = 11 ms, effective TE = 55 ms, 2 averages, 1 mm slice thickness, 9 slices, 20 × 20 mm^2^ FOV, 256 × 256 matrix, RARE factor = 12, acquisition time = 1:45 min:s) was acquired. The slice with the greatest mean tumor diameter was chosen for the subsequent single-slice sequences. T1 mapping was performed before and after injection of the contrast agent using a single-slice RARE sequence with variable TR (RARE-VTR, TR = 7500, 5000, 3000, 1500, 800, 400, 311, 123 ms, TE = 50 ms, 1 average, 1 mm slice thickness, 18 × 15 mm^2^ FOV, 108 × 96 matrix, acquisition time = 14:39 min:s) with consecutive quantification of T1 relaxation times using the image sequence analysis tool within ParaVision. For DCE imaging, the blood pool contrast agent gadofosveset trisodium (Lantheus Medical Imaging, North Billerica, Massachusetts, USA) was injected via a tail vein catheter (Klinika Medical GmbH, Usingen, Germany) with a concentration of 0.6 mmol/kg, using a perfusion pump (World Precision Instruments, Sarasota, Florida, USA) at a rate of 240 µL/min. The injection was initiated one minute after starting a FLASH (Fast Low Angle Shot) scan (TR = 24.6 ms, TE = 1.5 ms, 15° flip angle, 1 average, 610 repetitions, 18 × 15 mm^2^ FOV, 96 × 96 matrix, acquisition time = 20:00 min:s). As gadofosveset is a macromolecular contrast agent that binds reversibly to serum albumin and can thus only extravasate in case of enhanced vascular leakage, it enables not only assessment of tumor perfusion but also of endothelial permeability. Overall acquisition time of the entire MRI protocol was 51:03 min:s.

T2-weighted images were used to evaluate macroscopic changes after therapy and enabled assessment of tumor volume, analyzed using 3D Slicer (version 4.11.2021, https://www.slicer.org/). For subsequent analysis of tumor perfusion and permeability parameters, regions of interest (ROIs) were drawn around the viable tumor periphery with exclusion of necrotic tumor areas, using T2-weighted images as guidance. Three representative, non-overlapping ROIs were placed in the viable tumor periphery, and their mean yielded the average value per animal. To eliminate interindividual differences in pre-contrast T1 relaxation times, ROIs were copied from pre- to post-contrast T1 maps and delta of T1 values (*∆*T1) before and after contrast agent injection were calculated. Dynamic assessment of contrast enhancement after gadofosveset injection was used to derive the parameters area under the curve (*AUC*), maximum slope (slope_max_) and volume transfer constant *K*_trans_. While the area under the signal intensity curve and its maximum slope describe characteristics of tumor perfusion, the transfer constant *K*_trans_ assesses capillary permeability [36]. Furthermore, plasma volume fraction *v*_*p*_, a marker for vascularization, was calculated. Calculation of these parameters was performed with an in-house developed software based on the Pk modeling extension for 3D Slicer (https://github.com/millerjv/PkModeling). First, the acquired pre-contrast T1 map was resampled to the matrix and pixel size of the DCE-MRI using a linear interpolator. Subsequently, pharmacokinetic modeling of the DCE measurement was performed using a three-parameter Tofts model (also referred to as extended Tofts model) [37] and the parameters (*AUC*, slope_max_ and *K*_trans_) were calculated pixel by pixel. A population-based arterial input function was used [38]. The longitudinal relaxivity of the applied contrast agent was set to 0.0045 ms^−1^ mM^−1^, determined by extrapolation from literature values [39], and the hematocrit value was assumed to be 0.45.

### Histology and immunohistochemistry

For histological analysis, tumors were paraffin-embedded and sectioned in 5 µm tissue slices using a rotary microtome (Leica Microsystems, Nussloch, Germany). Hematoxylin and eosin (H&E) staining was performed according to standard protocols and enabled identification of intratumoral hemorrhage and necrosis. Immunohistochemical staining of CD31, a marker for vessel density and angiogenesis [40], was performed as previously described [41]. In brief, it was started with dewaxing and rehydration, followed by incubation in 3% H_2_O_2_ for 10 min. Afterward, sections were incubated with primary antibody CD31 (ab124432, Abcam, Cambridge, UK) for one hour at 4 °C in a dilution of 1:200 in blocking buffer, followed by horseradish peroxidase/diaminobenzidine detection. To analyze changes of the intratumoral immune cell infiltrate after immune checkpoint blockade, further immunohistochemical staining of CD3 (T-cells), F4/80 (macrophages), Ly6G (neutrophils), CD49b (natural killer cells) and CD45R (B-cells) was performed (Additional file 1: Meth. 1). For each group, *n* = 3 samples have been analyzed qualitatively.

### Transmission electron microscopy

Transmission electron microscopy enabled assessment of endothelial junction integrity and intactness of endothelial layers and basal laminae. Tumors were fixed in 2% (v/v) formaldehyde and 2.5% (v/v) glutaraldehyde in 100 mM cacodylate buffer. After washing in PBS, post-fixation was conducted with 0.5% (v/v) osmium tetroxide and 1% (w/v) potassium hexacyanoferrate (III) in 0.1 M cacodylate buffer. After washing in distilled water and dehydration in an ascending ethanol series, tumors were incubated in propylenoxide twice for 15 min each. Next, small tissue pieces (1–3 mm^3^) of the tumor border were embedded in Epon (Sigma-Aldrich, St. Louis, MO, USA) using flat embedding molds. 80 nm sections were sectioned using a ultramicrotome, collected on copper grids and negatively stained with 2% uranyl acetate for 15 min [42]. Electron micrographs were taken at a Philips EM-410 electron microscope (Philips, Amsterdam, The Netherlands). For each group, two samples have been analyzed qualitatively.

### Statistical analysis

Shapiro–Wilk test was used to check for normal distribution of data (*α* = 0.05). Depending on *p*-values, treatment groups were compared to untreated control groups using a two-tailed t-test or Mann–Whitney U test. Statistics were performed using SAS (version 9.4, Copyright SAS Institute Inc., Cary, NC, USA), and graphs were created using GraphPad Prism (version 9.2.0, GraphPad Software Inc., San Diego, California, USA). Group sizes were as followed: *n* = 12 for 67NR 6d untreated, *n* = 8 for 67NR 6d sorafenib, *n* = 8 for 67NR 6d ICI, *n* = 10 for 67NR 9d untreated, *n* = 8 for 67NR 9d sorafenib, *n* = 7 for 67NR 9d ICI, *n* = 12 for 4T1 6d untreated, *n* = 6 for 4T1 6d angiopoietin-1, *n* = 8 for 4T1 6d sorafenib, *n* = 8 for 4T1 6d ICI, *n* = 9 for 4T1 9d untreated, *n* = 8 for 4T1 9d sorafenib, n = 7 for 4T1 9d ICI. Descriptive statistics and further details for analysis of significance are provided in Additional file 1: Tables S1–S4.

## Results

### Angiopoietin-1-induced vessel stabilization

To evaluate if the MRI protocol allows accurate assessment of vessel stabilization, 4T1 tumor-bearing mice were treated with angiopoietin-1.

**Fig. 1:**
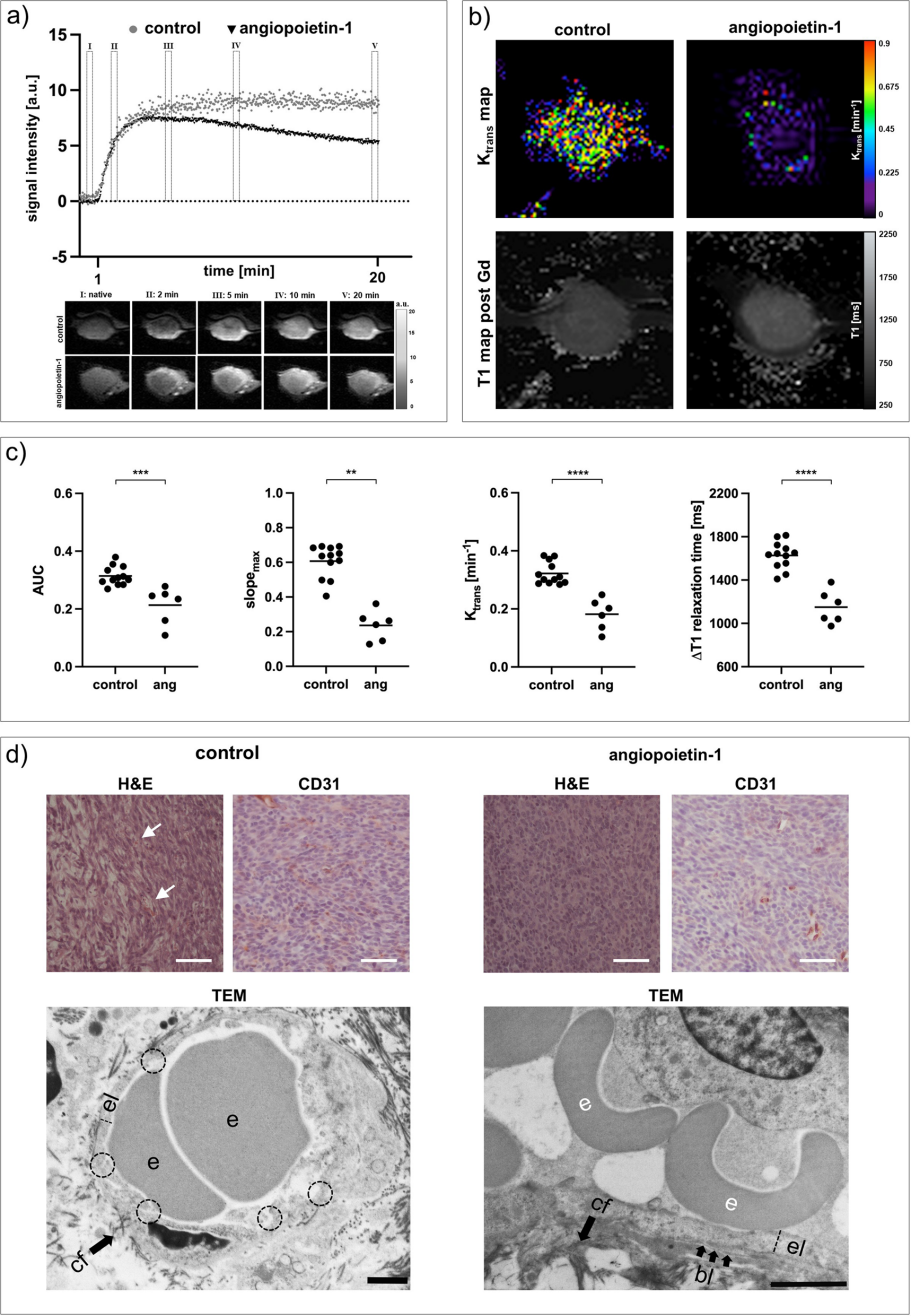
Analysis of vessel-stabilizing angiopoietin-1 treatment. **a** Exemplary contrast enhancement curves and corresponding sequential T1-weighted images of angiopoietin-1 treated tumors compared to untreated control tumors. While leaky 4T1 control tumors exhibit a plateau, angiopoietin-1 treated 4T1 tumors show a pronounced washout of the contrast agent. **b** Exemplary K_trans_ and T1 maps post-gadofosveset injection. **c** Area under the curve (AUC), maximum slope (slope_max_), K_trans_ and ∆T1 analyses assessing decreased tumor perfusion and permeability after treatment. Each dot represents one animal, with horizontal lines indicating the mean. **d** Ex vivo analysis of angiopoietin-1 treated tumors compared to control groups. While untreated 4T1 tumors show intratumoral hemorrhage (H&E, arrows), no hemorrhage is observed after angiopoietin-1 treatment, with tumors showing decreasing CD31 expression. Scale bars of immunohistochemical stainings represent 50 µm. Transmission electron micrographs (TEM) show thin endothelial layers with several interruptions (dashed circles) in untreated tumors. Angiopoietin-1 treated tumors demonstrate overall thicker and continuous endothelium with intact basal lamina. *e* erythrocyte, *el* endothelial layer, *bl* basal lamina, *cf* collagen fibrils. Scale bars of TEM images indicate 1 µm. **p < 0.01, ***p < 0.001, ****p < 0.0001

Angiopoietin-1 treatment had no significant influence on tumor volume compared to untreated control tumors on day six. However, sequential T1-weighted images and corresponding contrast enhancement curves of angiopoietin-1 treated tumors showed a much more pronounced wash-out of the contrast agent compared to untreated controls (Fig. 1a). All calculated tumor perfusion and permeability parameters, including area under the curve (*AUC*), maximum slope (*slope*_*max*_) and *K*_trans_, decreased significantly after treatment. In line, angiopoietin-1 treatment also reduced the intratumoral retention of gadofosveset, assessed by decreasing *∆T1* compared to untreated controls (Fig. 1b, c). Furthermore, plasma volume fraction *v*_*p*_, a marker for overall vascularization, decreased significantly after treatment of 4T1 tumors (Additional file 1: Fig. S1). Ex vivo analysis of tumor vasculature confirmed the in vivo imaging results qualitatively: While H&E staining showed intratumoral hemorrhage in untreated tumors, angiopoietin-1 treated tumors exhibited almost no intratumoral hemorrhage and the expression of CD31 was reduced. In addition, ultrastructural analysis of blood vessels using transmission electron microscopy confirmed improved integrity of the endothelial layer after angiopoietin-1 treatment: Compared to the thin and disrupted endothelium of untreated tumors, the angiopoietin-1 treated endothelium was overall thicker, with almost no endothelial gaps and an intact basal lamina (Fig. 1d).

### Impact of sorafenib treatment on tumor growth and vasculature

**Fig. 2:**
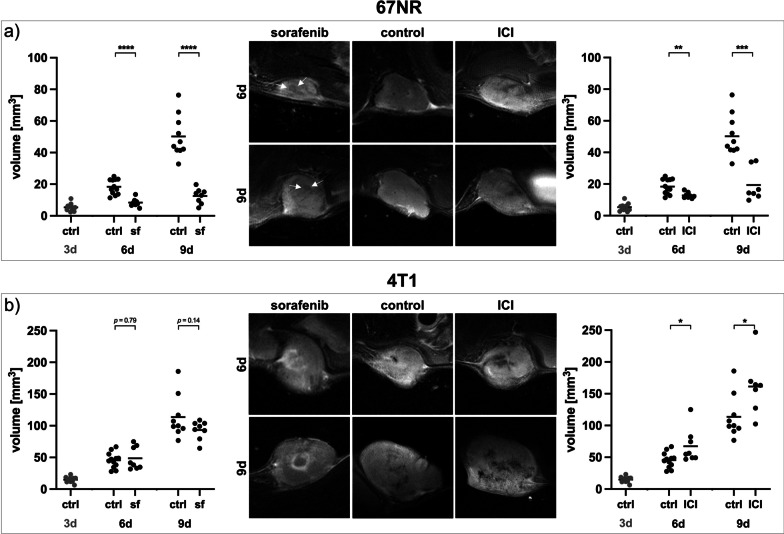
T2-weighted imaging and tumor volumetry after targeted therapy. Treatment initiation of both targeted therapies was started on day three (3d) after tumor implantation. On day six (6d) or day nine (9d), sorafenib and ICI-treated tumors were compared to untreated controls. **a** Size of 67NR tumors decreased after both sorafenib treatment and immune checkpoint blockade on day six and day nine, with small areas of intratumoral necrosis (white arrows). Tumor volumes of 67NR tumors before treatment initiation on day three are displayed in gray. **b** Sorafenib treatment caused no significant changes in volumes of 4T1 tumors both on day six and day nine. After treatment, tumors developed circumscribed necrosis, specifically arranged in the tumor center. ICI-treated 4T1 tumors showed increased tumor volumes compared to control groups and exhibited excessive intratumoral necrosis. Tumor volumes of 4T1 tumors before treatment initiation on day three are displayed in gray. Each dot represents one animal, with horizontal lines indicating the mean. ^*^p < 0.05, ^**^p < 0.01, ^***^p < 0.001, ^****^p < 0.0001

To assess the changes in tumor vasculature after antiangiogenic treatment, 4T1 or 67NR tumor-bearing mice were treated with the multi-kinase inhibitor sorafenib. Compared to untreated controls, sorafenib treatment resulted in a significantly reduced volume of 67NR tumors on both day six and day nine (Fig. 2a). However, sorafenib treatment had no significant effect on the volume of 4T1 tumors at any observed time point (Fig. 2b), with tumors showing pronounced hemorrhage in the tumor center.

**Fig. 3:**
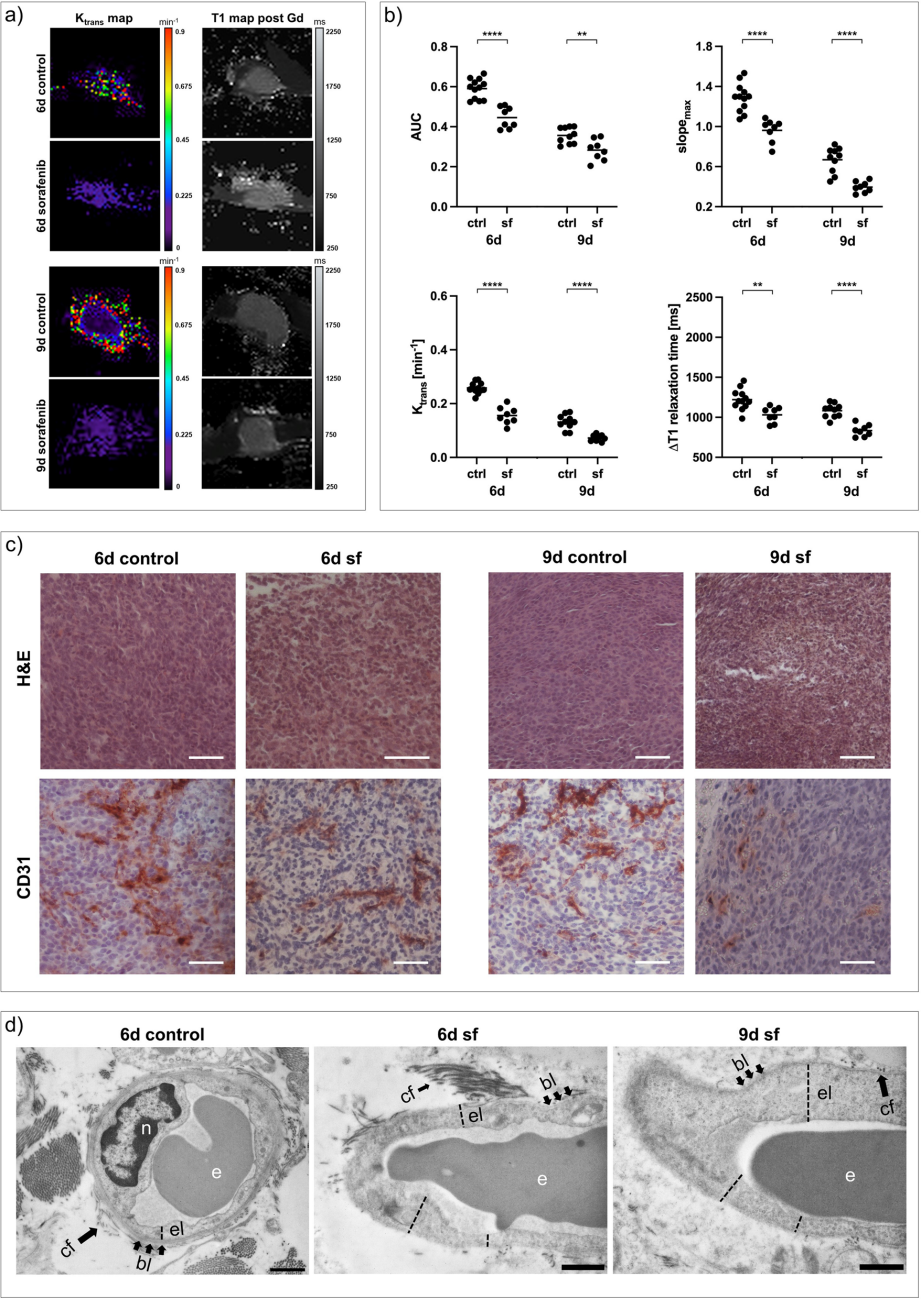
Changes in vasculature of 67NR tumors after sorafenib treatment. **a** Exemplary K_trans_ and T1 maps post-gadofosveset injection of sorafenib-treated 67NR tumors compared to untreated controls. **b** Area under the curve (AUC), maximum slope (slope_max_), K_trans_ and ∆T1 analyses indicating decreasing tumor perfusion and permeability parameters on day six and day nine. Each dot represents one animal, with horizontal lines indicating the mean. **c** H&E stainings of 67NR tumor sections show increasing areas of necrosis after treatment, while CD31 expression, especially on day nine, is reduced. Scale bars represent 50 µm. **d** Transmission electron micrographs (TEM) of 67NR tumors. Untreated 67NR tumors show a continuous endothelial layer with intact basal lamina. Sorafenib-treated tumors show similar characteristics with overall thicker endothelium. Scale bars represent 1 µm. *n* nucleus, *e* erythrocyte, *el* endothelial layer, *bl* basal lamina, *cf* collagen fibrils. ^**^p < 0.01, ^****^p < 0.0001

Anatomical T2-weighted images revealed small areas of necrosis in sorafenib-treated 67NR tumors compared to homogenous untreated controls. Sorafenib treatment of 67NR tumors resulted in significantly decreased tumor perfusion (*AUC*, slope_max_) and permeability (*K*_trans_) parameters with reduced retention of intratumoral gadofosveset, assessed by lower *∆T1* compared to untreated control group (Fig. 3a, b). In addition, *v*_*p*_ was reduced after treatment (Additional file 1: Fig. S1). In line with T2-weighted images, H&E staining of sorafenib-treated 67NR tumors showed small areas of necrosis, increasing from day six to day nine, which were not visible in untreated 67NR tumors. Again, qualitative ex vivo analysis of tumor vasculature validated the in vivo imaging results of reduced tumor perfusion and permeability. Compared to high CD31 expression of untreated 67NR tumors, CD31 expression decreased after treatment (Fig. 3c). Transmission electron microscopy of sorafenib-treated 67NR tumor vessels showed intact endothelial layers with a continuous basal lamina on both time points (Fig. 3d). In addition, in a separate set of experiments, mean gadolinium concentrations within the ex vivo tumor slices were quantified using laser ablation-inductively coupled plasma-mass spectrometry (LA-ICP-MS, Additional file 1: Meth. 2). The mean gadolinium concentrations showed a significant negative correlation with T1 relaxation times after gadofosveset injection (Additional file 1: Fig. S2). Since gadofosveset is a macromolecular, albumin-binding contrast agent, gadolinium extravasation and subsequent detection in LA-ICP-MS analysis occurs in tissues with increased endothelial permeability, confirming that the applied MRI approach can accurately assess vascular permeability.

**Fig. 4:**
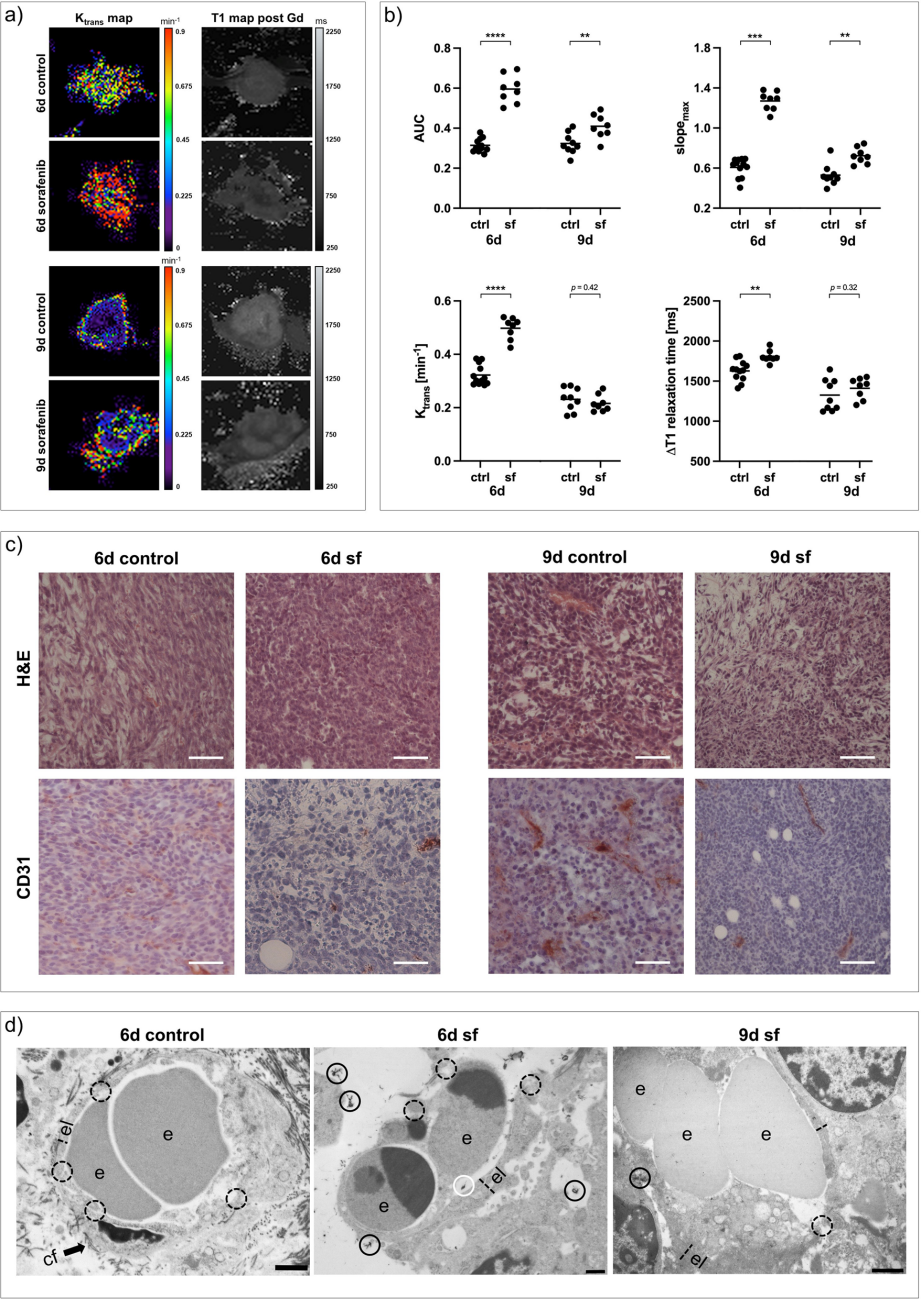
Changes in vasculature of 4T1 tumors after sorafenib treatment. **a** Exemplary K_trans_ and T1 maps post-gadofosveset injection of sorafenib-treated 4T1 tumors compared to untreated controls. **b** Area under the curve (AUC), maximum slope (slope_max_), K_trans_ and ∆T1 analyses indicating increasing tumor perfusion and permeability parameters on day six, with a consecutive decrease to day nine. Each dot represents one animal, with horizontal lines indicating the mean. **c** H&E staining of 4T1 tumor sections showing increasing areas of necrosis after treatment, while CD31 expression is reduced. Scale bars represent 50 µm. **d** Transmission electron micrographs (TEM) of 4T1 tumors. Untreated 4T1 tumors show a thin endothelial layer with short interruptions (dashed circles). After sorafenib treatment, endothelial layers are overall more intact with less interruptions (dashed circles). Electron dense contrast agent is visible intravasal (white circle) and extravasal (black circles), with reduced extravasated contrast agent from day six to day nine. Scale bars represent 1 µm. *e* erythrocyte, *el* endothelial layer, *bl* basal lamina, *cf* collagen fibrils. ^**^p < 0.01, ^***^p < 0.001, ^****^p < 0.001

In sorafenib-treated 4T1 tumors, T2-weighted images revealed increasing intratumoral hemorrhage and necrosis, specifically arranged as a round structure in the tumor center. Tumor vascularization marker *v*_*p*_ was reduced on both day six and day nine compared to control groups (Additional file 1: Fig. S1). However, tumors exhibited significantly increased tumor perfusion and permeability parameters early on day six (about 1.5-fold), with significantly elevated *∆T1* compared to untreated control group. From day six to nine, all DCE-derived perfusion and permeability parameters decreased. On day nine, tumor perfusion parameters (*AUC*, slope_max_) were still slightly elevated compared to control groups, while tumor permeability parameter *K*_trans_ as well as *∆*T*1* showed no significant differences as compared to control groups anymore (Fig. 4a, b). The specific tumor architecture of sorafenib-treated 4T1 tumors, including the round area of necrosis and hemorrhage, was also visible in H&E staining and LA-ICP-MS (Additional file 1: Fig. S3). In contrast to DCE-derived perfusion parameters being elevated compared to control groups on day six, CD31 expression of 4T1 tumors was reduced after treatment on both day six and day nine, as assessed qualitatively (Fig. 4c). While in vivo DCE-MRI assessed increasing permeability on day six, sorafenib-treated 4T1 tumors showed less interruptions of the endothelial layer compared to control groups on both time points (Fig. 4d).

### Impact of immune checkpoint blockade on tumor growth and vasculature

To further evaluate the effects of immunotherapy on tumor vasculature, 4T1 or 67NR tumor-bearing mice were treated with a combination of anti-PD1 and anti-CTLA4 immune checkpoint inhibitors. While size of ICI-treated 67NR tumors was significantly reduced on day six and day nine (Fig. 2a), ICI combination therapy led to a significant increase in size of 4T1 tumors as compared to control groups (Fig. 2b). Both tumor models exhibited substantial infiltration of T-cells after ICI therapy, as assessed by immunohistochemistry (Additional file 1: Fig. S4).

**Fig. 5:**
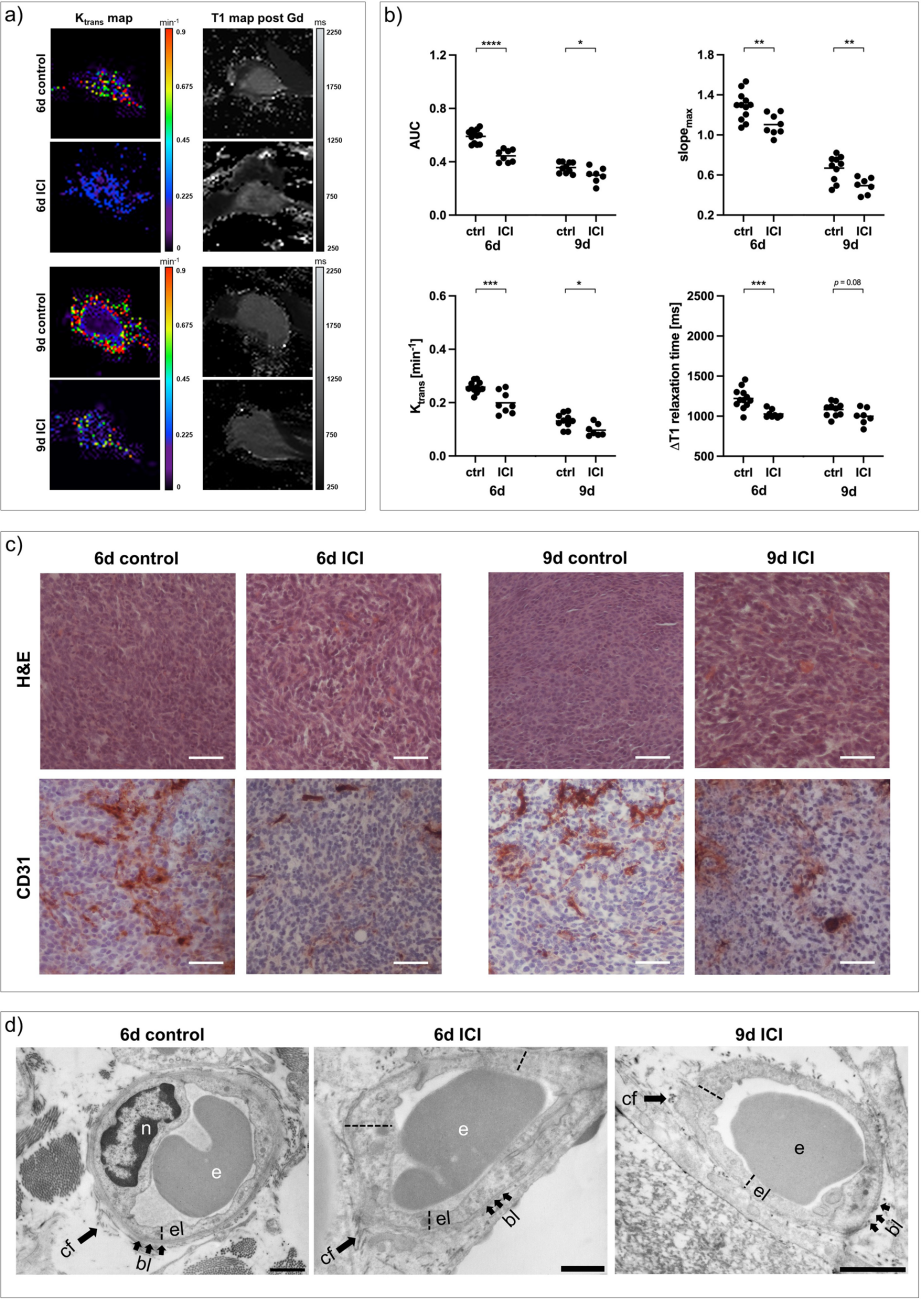
Changes in vasculature of 67NR tumors after ICI treatment. **a** Exemplary K_trans_ and T1 maps post-gadofosveset injection of ICI-treated 67NR tumors compared to untreated controls. **b** Area under the curve (AUC), maximum slope (slope_max_), K_trans_ and ∆T1 analyses indicating decreasing tumor perfusion and permeability parameters on day six and day nine. Each dot represents one animal, with horizontal lines indicating the mean. **c** H&E stainings of 67NR tumor sections show areas of intratumoral hemorrhage after treatment, which was not visible in control tumors. CD31 expression is reduced. Scale bars represent 50 µm. **d** Transmission electron micrographs (TEM) of 67NR tumors. Untreated 67NR tumors show a continuous endothelial layer with intact basal lamina. Also ICI-treated tumors show intact endothelial layers with apparently thicker endothelium. Scale bars represent 1 µm. *e* erythrocyte*, el* endothelial layer*, bl* basal lamina*, cf* collagen fibrils. ^*^p < 0.05, ^**^p < 0.01, ^***^p < 0.001, ^****^p < 0.0001

ICI-treated 67NR tumors showed only little intratumoral necrosis compared to untreated controls in T2-weighted imaging. Similar to sorafenib treatment, all DCE-derived perfusion and permeability parameters decreased, paralleled by lower intratumoral gadolinium retention (Fig. 5a, b) and reduction in vascularization marker *v*_*p*_ (Additional file 1: Fig. S1). In line with in vivo imaging results, H&E staining of ICI-treated 67NR tumors showed small areas of necrosis with little intratumoral hemorrhage, while qualitatively assessed CD31 expression of 67NR tumors decreased after treatment (Fig. 5c). Transmission electron microscopy revealed intact and thick endothelium with a continuous surrounding basal lamina, again similar to the characteristics after antiangiogenic sorafenib treatment (Fig. 5d).

**Fig. 6:**
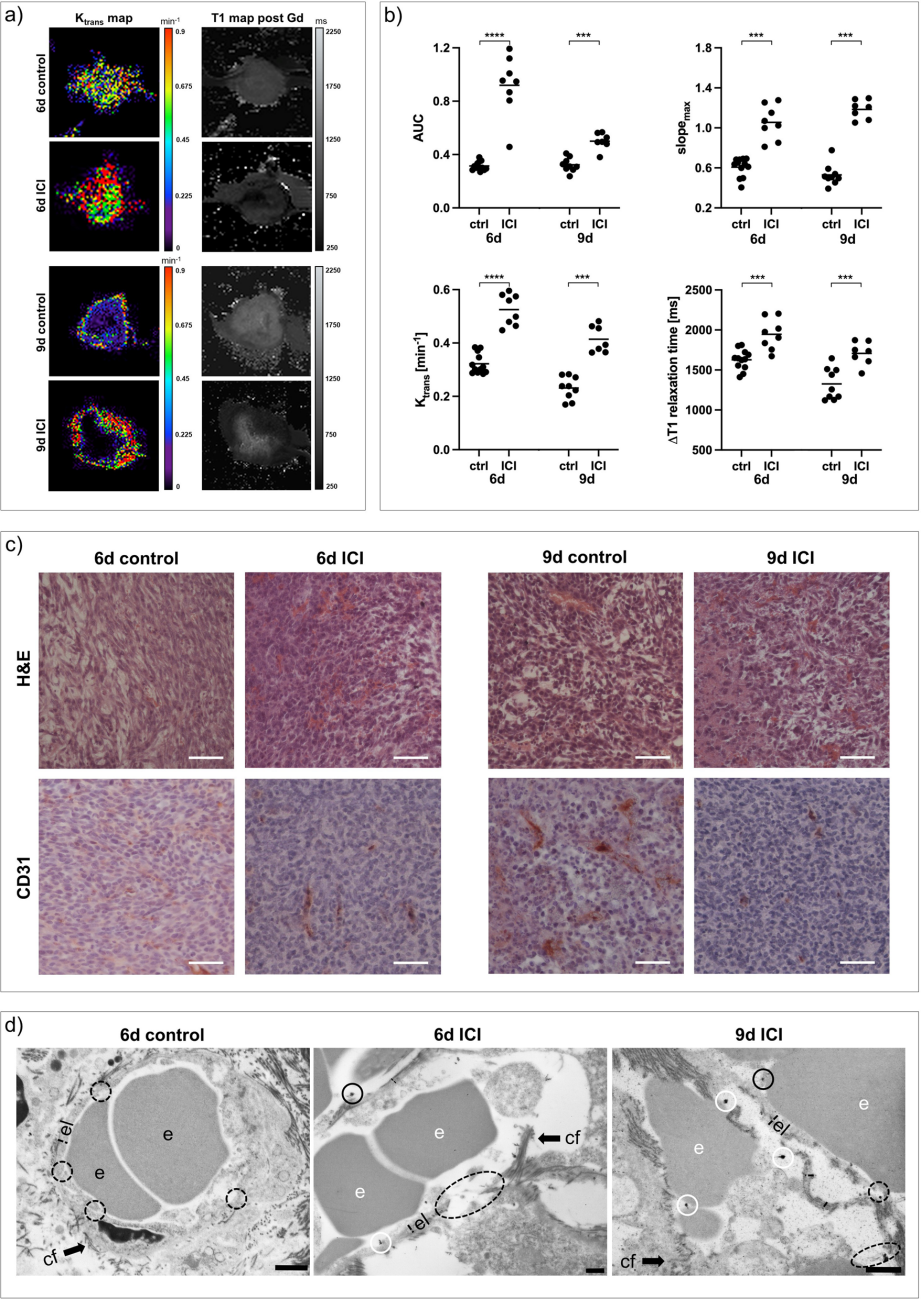
Changes in vasculature of 4T1 tumors after ICI treatment. **a** Exemplary K_trans_ and T1 maps post-gadofosveset injection of ICI-treated 4T1 tumors compared to untreated controls. **b** Area under the curve (AUC), maximum slope (slope_max_), K_trans_ and ∆T1 analyses indicating increasing tumor perfusion and permeability parameters on day six and day nine. Each dot represents one animal, with horizontal lines indicating the mean. **c** H&E stainings of 4T1 tumor sections showing strongly increasing intratumoral hemorrhage after treatment. CD31 expression is reduced. Scale bars represent 50 µm. **d** Transmission electron micrographs (TEM) of 4T1 tumors. Untreated 4T1 tumors show a thin endothelial layer with short interruptions (dashed circles). After ICI treatment, endothelial layers are thin, showing very large interruptions (dashed ovals). Electron dense contrast agent is visible intravasal (white circle) and extravasal (black circles). Scale bars represent 1 µm. *e* erythrocyte, *el* endothelial layer, *bl* basal lamina, *cf* collagen fibrils. ^***^p < 0.001, ^****^p < 0.0001

In contrast, ICI-treated 4T1 tumors exhibited elevated DCE-derived perfusion and permeability parameters with elevated gadolinium retention after treatment (Fig. 6a, b), accompanied by decreased vascularization (Additional file 1: Fig. S1). Even though qualitatively assessed CD31 expression decreased, H&E staining showed excessive intratumoral hemorrhage and increasing tumor necrosis. Transmission electron micrographs confirmed enhanced vascular leakage, by capturing very thin and tortuous endothelial layers with markedly increasing interruptions and endothelial cells being detached from the basal lamina after treatment (Fig. 6d).

## Discussion

The aim of this study was to noninvasively assess and compare the vascular response patterns to two different targeted therapies (sorafenib or immune checkpoint inhibitors) between highly malignant 4T1 and low malignant 67NR tumors using dynamic contrast-enhanced MRI.

Conventional chemotherapy targets proliferating cells unselectively, and its successful antitumor effects can be reliably detected by decreasing lesion size. In comparison, targeted therapies alter specific characteristics of the tumor microenvironment, including tumor perfusion and permeability. These changes frequently occur without an immediate effect on lesion size; thus, response assessment becomes more challenging [43]. Indeed, tumor volumetry, performed using T2-weighted images, was not suitable to reliably indicate a therapy response in this study either. Although low malignant 67NR tumors demonstrated a reduction in tumor size after both targeted therapies, this was not the case for the highly malignant 4T1 model, which showed no significant changes in tumor size after sorafenib treatment and even increasing size after immune checkpoint blockade, compared to control groups.

Instead, this study clearly demonstrates that DCE-MRI with an albumin-binding contrast agent is able to capture early changes in tumor vasculature after both antiangiogenic treatment and immune checkpoint blockade, while revealing different response patterns between tumors with varying grade of malignancy. Low malignant 67NR tumors showed decreasing tumor perfusion and permeability after sorafenib treatment, while 4T1 tumors were characterized by an initial increase in DCE-derived parameters on day six, with a subsequent decrease to day nine. While ICI therapy also led to decreased tumor perfusion and permeability in 67NR tumors, parameters strongly increased in ICI-treated 4T1 tumors. The suitability of the DCE-MRI approach with albumin-binding gadofosveset to evaluate both tumor perfusion and vessel permeability was indicated by reduced perfusion and permeability parameters after angiopoietin-1 treatment, which is known for its vessel-stabilizing effects [26]. Furthermore, the vascular changes implied by noninvasive DCE-MRI were confirmed by ex vivo structural analysis of tumor blood vessels using histology and transmission electron microscopy, providing evidence that the calculated DCE-MRI parameters truly reflect tumor vasculature characteristics.

In low malignant 67NR tumors, sorafenib treatment was able to inhibit angiogenesis and stabilize intratumoral blood vessels. In line with immunohistochemistry showing a reduced CD31 expression and electron microscopy revealing intact and continuous endothelial layers of tumor capillaries, DCE imaging parameters confirmed decreasing tumor perfusion and vessel permeability after treatment. These vascular changes after antiangiogenic therapy have already been recorded in other preclinical and clinical studies [24, 25, 44, 45]. They are in accordance with the initial rationale that sorafenib inhibits blood vessel formation, which in turn will cause profound vascular regression and perfusion, leading to tumors ‘starving to death’ [46, 47]. Using DCE-MRI, these therapy-induced effects can be monitored noninvasively and longitudinally over time. Compared to 67NR tumors, the effects of sorafenib treatment on vasculature of highly malignant 4T1 tumors differed strongly. On day three after treatment initiation, sorafenib-treated 4T1 tumors exhibited increasing tumor perfusion and permeability compared to control groups. Three days later, perfusion and permeability decreased to values almost comparable with control groups again. These early vascular changes after antiangiogenic treatment indicate a stabilization of the vessel barrier [48]. By reverting tumor vasculature from a grossly abnormal structure toward a more physiological state, antiangiogenic therapies can create a transient window of enhanced tumor perfusion and oxygenation for a few days, followed by subsequent reduction in perfusion [47–49]. While we were able to capture vascular normalization on treatment day three, followed by decreasing parameters on day six, we are not able to draw conclusions about the potential long-term changes beyond this period. Increases of *K*_trans_ values in the 4T1 model have already been shown after bevacizumab, another antiangiogenic treatment [50]. In recent studies, the vascular normalization window after antiangiogenic therapy and its subsequent enhanced tumor perfusion was used to improve the drug delivery of other therapies, e.g., chemotherapies, into the tumor lesion [11]. However, as it is only a transient stage of improved tumor perfusion, it is important to be able to precisely detect these vascular changes, which is possible using DCE-MRI as presented. In this study, the vascular normalization phenomenon was only visible in the highly malignant 4T1 model, as only these tumors were initially characterized by a tortuous and abnormal tumor vasculature, while the vascular system of low malignant 67NR tumors was generally more intact at baseline before treatment [31].

The response patterns of both tumor models did not only differ after sorafenib treatment but also after immune checkpoint blockade. In the low malignant 67NR model, ICI treatment also mediated stabilizing effects on intratumoral blood vessels with decreasing tumor perfusion and permeability, in line with reduction in CD31 expression and microstructurally intact blood vessels. These therapeutic effects after ICI treatment have also been observed in other preclinical studies [51, 52], applied to different murine breast cancer models and a fibrosarcoma model, as well as first clinical studies in patients with metastatic melanoma [13]. They may be attributed to vascular remodeling after cytokine secretion of tumor-infiltrating immune cells, such as TNF*α* [52]. Contrary to that, highly malignant 4T1 tumors exhibited enhanced DCE-derived perfusion and permeability parameters with overall slightly increasing CD31 expression. In this context, therapy-induced hyperperfusion and enhanced endothelial permeability were recently found to be correlated with successful activation of antitumor T-cell immunity [15, 53], which again underlines that the response patterns after targeted therapies are marked by huge intra- and intertumoral heterogeneity.

The divergent response patterns to both targeted therapies between the two tumor models used within this study might be related to differences in a variety of tumor characteristics determined by their respective malignant potential. While low malignant 67NR tumors demonstrated overall maturity and integrity of their vascular system, rapid tumor growth of highly malignant 4T1 tumors led to abnormal and tortuous intratumoral blood vessels, hindering drug delivery into the tumor lesion. The dominating intratumoral immune cell populations and their secretion of different angiogenic factors as well as PD-L1 expression of the tumors might additionally contribute to the divergent vascular response patterns [15, 54]. Furthermore, different response patterns might be attributable to differences in mutational status, epigenetic modification and cancer cell plasticity between tumors with varying malignant potential [55].

Despite the presented accuracy for detecting different vascular response patterns between both tumor models, the presented study is limited in some aspects of methodology. Due to the high temporal resolution, only single-slice MR imaging was performed, which led to loss of information on slice-adjacent tumor areas. However, multi-slice T2-weighted imaging was used to place DCE slices across the largest tumor diameter, suggesting representative tumor areas and reducing partial volume effects. ROIs were placed around the viable tumor border to exclude tumor necrosis, where perfusion parameters do not lead to meaningful conclusions about tumor vasculature. For future studies, reduction in temporal in favor of spatial resolution could be performed to further assess intratumoral heterogeneity, a main contributor to malignant tumor progression, metastasis formation and therapy resistance [56]. For precise analysis of not only tumor perfusion but also permeability, numerous studies have demonstrated that macromolecular contrast agents (MMCA) are more favorable than small molecular contrast agents (SMCA) [57–59]. While SMCAs have an unselective extracellular extravasation profile and already extravasate in physiological tissue, MMCAs follow an intravascular distribution profile and are only able to extravasate when the endothelial permeability is pathologically enhanced, as known in tumor vasculature. Due to the use of MMCAs, DCE-MRI analysis using two-compartment, unidirectional pharmacokinetic models, which neglect the efflux of the contrast agent from the interstitial space back to intravascular space, could potentially lead to altered results when compared to the two-compartment, bidirectional extended Tofts model used within this study [60]. Instead of longitudinal measurements, mice were killed after each time point to enable ex vivo analysis of tumor tissue. Since it is very difficult to compare the exact same ROIs in histology and MRI, ex vivo analysis is able to validate the key findings of in vivo DCE-MRI, but no direct correlations between histology and MRI can be drawn. While this study performed qualitative ex vivo analysis of tumor tissue, further quantitative ex vivo analysis would enable stronger validation of in vivo MRI data. In this regard, immunohistochemical analysis of tumor vasculature could be extended to αSMA for more detailed analysis of vessel maturity. The initial increase in size of 4T1 tumors after ICI therapy may suggest pseudoprogression. However, to validate a subsequent decrease in tumor size and to assess long-term therapy response, tumors would have had to be observed for a longer period of time including overall survival analyses, which was not possible due to the animal protocol.

Divergent trends in treatment responses were observed between the highly malignant 4T1 and low malignant 67NR tumor model. To further validate that the differing response patterns observed in this study are caused by the divergent degrees of malignancy, the study should be expanded to other tumor entities. It should ideally also include a direct comparison of responding and non-responding lesions.

## Conclusion

In summary, DCE-MRI was able to noninvasively assess different vascular response patterns to treatment with the multi-kinase inhibitor sorafenib or immune checkpoint blockade between low malignant and highly malignant tumors. By capturing early changes of tumor vasculature, a key component of the tumor microenvironment, it may allow for personalized treatment decisions, with the possibility to guide early therapeutic decisions in patients that fail fast.

## Supplementary Information

**Additional file 1 Method 1:** Immunohistochemical analysis of the intratumoral immune cell infiltrate. **Method 2:** Laser ablation-inductively coupled plasma-mass spectrometry. **Fig. S1:** Analysis of plasma volume fraction vp. **Fig. S2:** Correlation between T1 relaxation times after contrast agent injection and LA-ICP-MS analysis. **Fig. S3:** Architecture of sorafenib-treated 4T1 tumors. **Fig. S4: **Immunohistochemical analysis of the intratumoral immune cell infiltrate after immune checkpoint blockade. **Table S1:** Descriptive statistics of 67NR tumors. **Table S2:** Analysis of significance of 67NR tumors. **Table S3: **Descriptive statistics of 4T1 tumors. **Table S4:** Analysis of significance of 4T1 tumors.

## Data Availability

The datasets generated during and/or analyzed during the current study are available from the corresponding author on reasonable request.

## Abbreviations

AUC: Area under the curve
DCE: Dynamic contrast-enhanced
FLASH: Fast low angle shot
FOV: Field of view
H&E: Hematoxylin and eosin
ICI: Immune checkpoint inhibitors
*K*_trans_: Volume transfer constant
LA-ICP-MS: Laser ablation-inductively coupled plasma-mass spectrometry
MMCA: Macromolecular contrast agents
MRI: Magnetic resonance imaging
RARE: Rapid acquisition with relaxation enhancement
VTR: Variable repetition time
ROI: Region of interest
slope_max_: Maximum slope
SMCA: Small molecular contrast agents
TEM: Transmission electron microscopy
TME: Tumor microenvironment
TE: Echo time
TR: Repetition time
