# Supplementary material for: Vascular response patterns to targeted therapies in murine breast cancer models with divergent degrees of malignancy
Source: Breast Cancer Res. 2023 May 23;25:56. doi: 10.1186/s13058-023-01658-9 (PMC10207609; doi:10.1186/s13058-023-01658-9)
Supplement: Supplementary file 1 — Additional file 1 Method 1: Immunohistochemical analysis of the intratumoral immune cell infiltrate. Method 2: Laser ablation-inductively coupled plasma-mass spectrometry. Fig. S1: Analysis of plasma volume fraction vp. Fig. S2: Correlation between T1 relaxation times after contrast agent injection and LA-ICP-MS analysis. Fig. S3: Architecture of sorafenib-treated 4T1 tumors. Fig. S4: Immunohistochemical analysis of the intratumoral immune cell infiltrate after immune checkpoint blockade. Table S1: Descriptive statistics of 67NR tumors. Table S2: Analysis of significance of 67NR tumors. Table S3: Descriptive statistics of 4T1 tumors. Table S4: Analysis of significance of 4T1 tumors. [file 13058_2023_1658_MOESM1_ESM.pdf]

Vascular response patterns to targeted therapies in murine breast cancer models with divergent degrees of malignancy – Supplementary material

Emily Hoffmann[1,*,#], Mirjam Gerwing[1,*], Tobias Krähling[1], Uwe Hansen[2], Katharina Kronenberg[3], Max Masthoff[1], Christiane Geyer[1], Carsten Höltke[1], Lydia Wachsmuth[1], Regina Schinner[4], Verena Hoerr[1,5], Walter Heindel[1], Uwe Karst[3], Michel Eisenblätter[1,6], Bastian Maus[1], Anne Helfen[1], Cornelius Faber[1], Moritz Wildgruber[1,4]

[1]University of Münster, Clinic of Radiology, Münster, Germany

[2]University of Münster, Institute for Musculoskeletal Medicine, Münster, Germany

[3]University of Münster, Institute of Inorganic and Analytical Chemistry, Münster, Germany

[4]Department of Radiology, University Hospital, LMU Munich, Munich, Germany

[5]Heart Center Bonn, Department of Internal Medicine II, University Hospital Bonn, Bonn, Germany

[6]Department of Diagnostic and Interventional Radiology, Medical Faculty OWL, University of Bielefeld, Bielefeld, Germany

[*]These authors contributed equally.

[#]Correspondence: emily.hoffmann@ukmuenster.de

Content

Suppl. Method 1:	Immunohistochemical staining of the intratumoral immune cell infiltrate.
Suppl. Method 2:	Laser ablation-inductively coupled plasma-mass spectrometry.
Suppl. Figure 1:	Analysis of plasma volume fraction v_p.
Suppl. Figure 2:	Correlation between T1 relaxation times after contrast agent injection and LA-ICP-MS analysis.
Suppl. Figure 3:	Architecture of sorafenib treated 4T1 tumors.
Suppl. Figure 4:	Immunohistochemical analysis of the intratumoral immune cell infiltrate after immune checkpoint blockade.
Suppl. Table 1:	Descriptive statistics of 67NR tumors.
Suppl. Table 2:	Analysis of significance of 67NR tumors.
Suppl. Table 3:	Descriptive statistics of 4T1 tumors.
Suppl. Table 4:	Analysis of significance of 4T1 tumors.

Suppl. Method 1: Immunohistochemical staining of the intratumoral immune cell infiltrate.

After deparaffinization and rehydration, tumor sections were incubated in blocking buffer (2% horse serum and 0.1% fetal calf serum in phosphate-buffered saline, PBS) for 45 minutes at room temperature. Afterwards, sections were incubated in a humidified chamber overnight at 4°C with primary antibodies against CD3 (rabbit, ab16669, abcam, Cambridge, UK), F4/80 (rat, BM4007S, Origene, Rockville, Maryland, USA), Ly6G (rat, 127602, Biolegend, San Diego, California), CD49b (rat, 108902, Biolegend) or CD45R (rat, 553084, BD Biosciences, Franklin Lakes, New Jeryey, USA), each at a dilution of 1:100 in blocking buffer. After washing the slides with PBS, incubation with the secondary antibodies anti-rabbit Alexa Fluor 488 (ab2536097, Thermo Fisher Scientific, Waltham, Massachusetts, USA) for CD3, anti-rat Alexa Fluor 647 (ab2535875) for F4/80 and anti-rat Alexa Fluor 594 (a21309, Invitrogen, Waltham, Massachusetts, USA) for Ly6G, CD49b and CD45R was performed for 60 minutes at room temperature. Nuclei were stained using 20 µM Hoechst 3342 dye (62249, Thermo Fisher Scientific) for 5 minutes, followed by washing with PBS.

Suppl. Method 2: Laser ablation-inductively coupled plasma-mass spectrometry.

To assess the intratumoral retention of gadolinium after gadofosveset injection, quantitative laser ablation-inductively coupled plasma-mass spectrometry (LA-ICP-MS) analysis using matrix-matched gelatin standards containing known concentrations of gadolinium (0 to 600 mg/L) was performed [1, 2]. In brief, frozen tumor samples and the gelatin standards were sectioned into 10 µm slices using a cryomicrotome (CryoStar™ NX70, Thermo Fisher, Bremen, Germany). Ablation of untreated control tumor slices was conducted as previously published [2]. For laser ablation of treated tumor slices, a line-by-line scan with a laser spot size of 15 µm, a repetition rate of 20 Hz, a He flow of 800 mL/min as transport gas and a scan speed of 45 µm/s was performed. The LSX 213 G2+ (CETAC Technologies, Omaha, Nebraska, USA) laser ablation system was directly connected via Tygon tubing to the 7900x ICP-MS (Agilent Technologies, Santa Clara, California, USA). Quantification and visualization of the analyzed elements were carried out with an in-house developed software (ImaJar 3.64, written by Robin Schmid). Similar to *in vivo* MRI data, ROIs were drawn around the viable tumor border to exclude necrotic tumor areas. For untreated control tumors, three samples with one section each were analyzed for both time points and cell lines. For therapy groups, one sample for each time point, cell line and therapy was analyzed. To compare T1 relaxation times after

gadofosveset injection to the gadolinium concentrations measured by means of LA-ICP-MS, corresponding Spearman rank correlation coefficient was calculated.

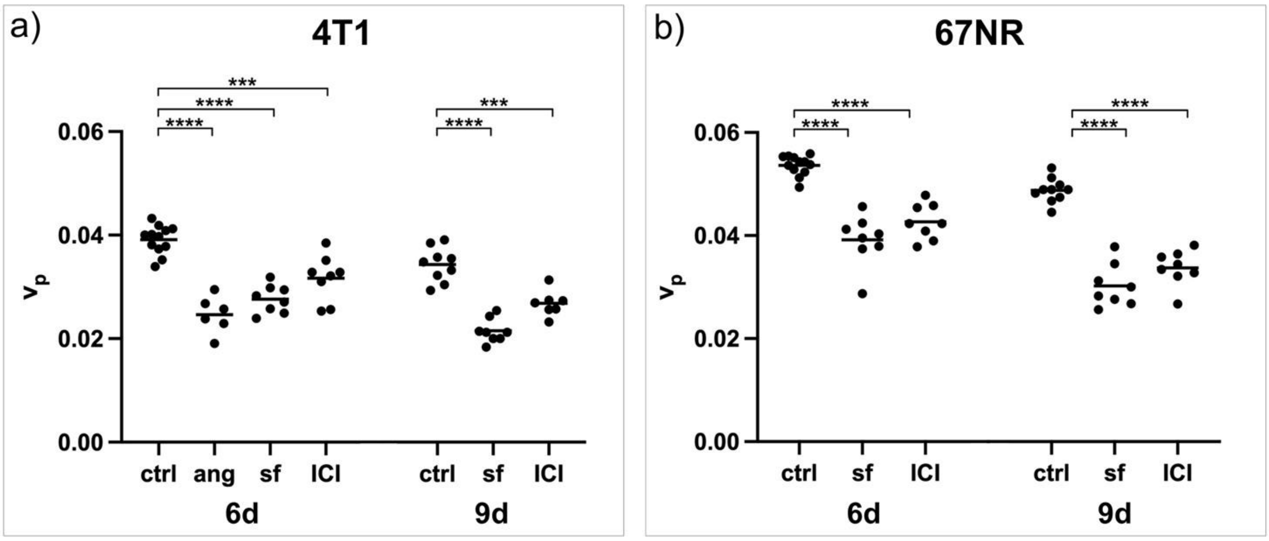

Suppl. Fig. 1: Analysis of plasma volume fraction v_p.
Plasma volume fraction v_p, a proposed DCE-derived parameter for vascularization [3], decreased significantly after angiopoietin-1 (ang), sorafenib (sf) and immune checkpoint inhibitor (ICI) treatment, both in **a)** 4T1 and **b)** 67NR tumors. $^{***}p < 0.001$, $^{****}p < 0.0001$

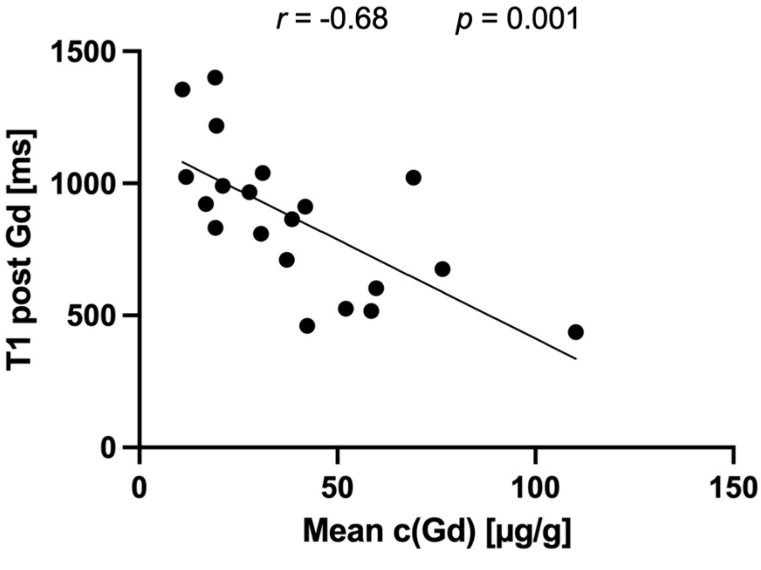

Suppl. Fig. 2: Correlation between T1 relaxation times after contrast agent injection and LA-ICP-MS analysis. Mean Gd concentrations of the tumor border, assessed by LA-ICP-MS, showed a significant negative correlation with T1 relaxation times after gadofosveset injection. Since gadofosveset is a macromolecular, albumin-binding contrast agent, gadolinium extravasation and subsequent detection in LA-ICP-MS analysis occurs in tissues with increased endothelial permeability, confirming that the applied MRI approach can accurately assess vascular permeability. The analysis includes $n = 12$ control and $n = 8$ treated tumors (Suppl. Meth. 1).

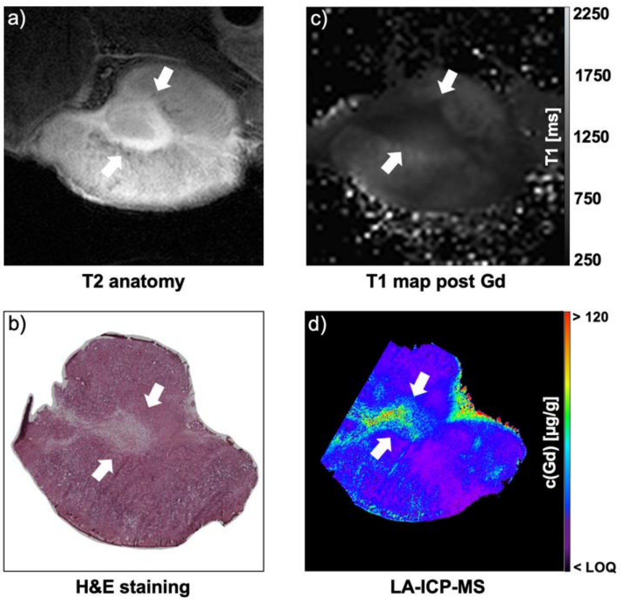

Suppl. Fig. 3: Architecture of sorafenib treated 4T1 tumors.
Six days after therapy initiation, sorafenib treated 4T1 tumors exhibited a circumscribed area of necrosis and hemorrhage, specifically arranged as a round structure in the tumor center, visible in **a**) T2-weighted imaging, **b**) H&E staining, **c**) T1 maps after gadofosveset injection and **d**) LA-ICP-MS. This architecture did not occur in sorafenib treated 67NR tumors.

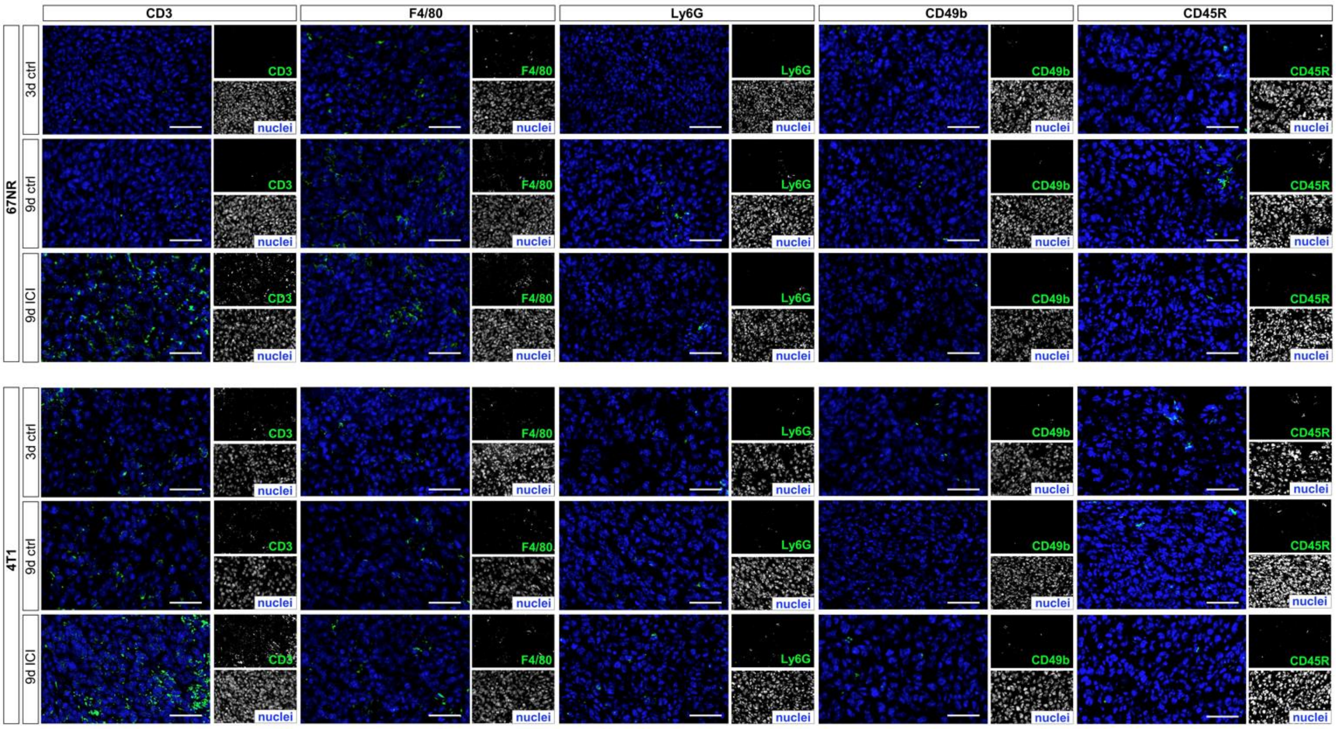

Suppl. Fig. 4: Immunohistochemical analysis of the intratumoral immune cell infiltrate after immune checkpoint blockade.
Representative 4T1 and 67NR tumor sections after 2-channel staining of nuclei and CD3 (T-cells), F4/80 (macrophages), Ly6G (neutrophils), CD49b (natural killer cells) or CD45R (B-cells) of untreated controls on day three (day of treatment initiation), untreated controls on day nine and ICI treated tumors on day nine. Immune checkpoint inhibition caused substantial intratumoral T-cell infiltration in both tumor models, with approximately constant proportions of the other examined immune cell populations. Scale bars represent 100 µm.

Suppl. Table 1: Descriptive statistics of 67NR tumors.
All values are presented as mean ± standard deviation.

Parameter	6d control	6d sf	6d ICI	9d control	9d sf	9d ICI
volume [mm^3]	18 ± 5	8 ± 3	13 ± 2	50 ± 13	13 ± 5	19 ± 10
AUC	0.59 ± 0.05	0.45 ± 0.05	0.44 ± 0.05	0.36 ± 0.04	0.28 ± 0.05	0.30 ± 0.06
$slope_{max}$	1.29 ± 0.14	0.96 ± 0.11	1.1 ± 0.11	0.67 ± 0.13	0.40 ± 0.06	0.49 ± 0.08
K_{trans} [min^{-1}]	0.26 ± 0.02	0.16 ± 0.03	0.20 ± 0.04	0.13 ± 0.03	0.07 ± 0.01	0.10 ± 0.02
ΔT1 [ms]	1220 ± 130	1030 ± 100	1030 ± 50	1080 ± 90	830 ± 70	1000 ± 100

Suppl. Table 2: Analysis of statistical significance of 67NR tumors.

Parameter	6d ctrl vs. sf		6d ctrl vs. ICI		9d ctrl vs. sf		9d ctrl vs. ICI	
	Shapiro Wilk *p*-value	t-test/Mann Whitney U *p*-value	Shapiro Wilk *p*-value	t-test/Mann Whitney U *p*-value	Shapiro Wilk *p*-value	t-test/Mann Whitney U *p*-value	Shapiro Wilk *p*-value	t-test/Mann Whitney U *p*-value
volume [mm^3]	0.16	< 0.0001	0.01	0.007	0.17	< 0.0001	0.39	0.0001
AUC	0.10	< 0.0001	0.03	< 0.0001	0.10	0.004	0.06	0.03
$slope_{max}$	0.57	< 0.0001	0.36	0.005	0.12	< 0.0001	0.28	0.006
K_{trans} [min^{-1}]	0.89	< 0.0001	0.66	0.0005	0.07	< 0.0001	0.13	0.01
ΔT1 [ms]	0.83	0.002	0.62	0.0008	0.74	< 0.0001	0.34	0.08

Suppl. Table 3: Descriptive statistics of 4T1 tumors.
All values are presented as mean ± standard deviation.

Parameter	6d control	6d ang	6d sf	6d ICI	9d control	9d sf	9d ICI
volume [mm^3]	47 ± 13	48 ± 14	49 ± 18	67 ± 27	110 ± 30	93 ± 15	160 ± 40
AUC	0.32 ± 0.03	0.21 ± 0.06	0.60 ± 0.07	0.92 ± 0.22	0.32 ± 0.05	0.41 ± 0.06	0.50 ± 0.06
$slope_{max}$	0.61 ± 0.09	0.24 ± 0.09	1.27 ± 0.09	1.06 ± 0.17	0.53 ± 0.11	0.72 ± 0.08	1.21 ± 0.14
K_{trans} [min^{-1}]	0.33 ± 0.04	0.18 ± 0.05	0.50 ± 0.04	0.53 ± 0.06	0.23 ± 0.04	0.22 ± 0.03	0.41 ± 0.05
ΔT1 [ms]	1630 ± 140	1150 ± 160	1810 ± 80	1950 ± 190	1330 ± 200	1410 ± 130	1710 ± 150

Suppl. Table 4: Analysis of statistical significance of 4T1 tumors.

Parameter	6d ctrl vs. ang		6d ctrl vs. sf		6d ctrl vs. ICI		9d ctrl vs. sf		9d ctrl vs. ICI	
	Shapiro Wilk *p*-value	t-test/Mann Whitney U *p*-value	Shapiro Wilk *p*-value	t-test/Mann Whitney U *p*-value	Shapiro Wilk *p*-value	t-test/Mann Whitney U *p*-value	Shapiro Wilk *p*-value	t-test/Mann Whitney U *p*-value	Shapiro Wilk *p*-value	t-test/Mann Whitney U *p*-value
volume [mm^3]	0.28	0.87	0.16	0.79	0.01	0.01	0.17	0.14	0.39	0.03
AUC	0.15	0.0008	0.01	< 0.0001	0.001	< 0.0001	0.006	0.007	0.002	0.0007
$slope_{max}$	0.13	0.001	0.01	0.0002	0.21	0.0002	0.02	0.003	0.001	0.0002
K_{trans} [min^{-1}]	0.32	< 0.0001	0.03	< 0.0001	0.04	< 0.0001	0.24	0.42	0.03	0.0002
ΔT1 [ms]	0.73	< 0.0001	0.27	0.004	0.93	0.0009	0.07	0.32	0.22	0.0008

References

[1] Gerwing M, Hoffmann E, Kronenberg K et al (2022) Multiparametric MRI enables for differentiation of different degrees of malignancy in two murine models of breast cancer. Front Oncol 12:1000036. https://doi.org/10.3389/fonc.2022.1000036

[2] Funke SKI, Factor C, Rasschaert M et al (2022) Long-term Gadolinium Retention in the Healthy Rat Brain: Comparison between Gadopiclenol, Gadobutrol, and Gadodiamide. Radiology 305:179–189. https://doi.org/10.1148/radiol.212600

[3] Keil VC, Gielen GH, Pintea B et al (2021) DCE-MRI in Glioma, Infiltration Zone and Healthy Brain to Assess Angiogenesis: A Biopsy Study. Clin Neuroradiol 31:1049-1058. https://doi.org/10.1007/s00062-021-01015-3
